# A Novel Ensemble Adaptive Sparse Bayesian Transfer Learning Machine for Nonlinear Large-Scale Process Monitoring

**DOI:** 10.3390/s20216139

**Published:** 2020-10-28

**Authors:** Hongchao Cheng, Yiqi Liu, Daoping Huang, Chong Xu, Jing Wu

**Affiliations:** 1 School of Automation Science and Engineering, South China University of Technology, Guangzhou 510640, China; Hongchao.Cheng@uts.edu.au (H.C.); audhuang@scut.edu.cn (D.H.); 0800079@gnnu.edu.cn (C.X.); auipicq@mail.scut.edu.cn (J.W.); 2 Centre for Technology in Water and Wastewater, School of Civil and Environmental Engineering, University of Technology Sydney, Ultimo, NSW 2007, Australia; 3 School of Data Science and Information Engineering, Guizhou Minzu University, Guiyang 550025, China

**Keywords:** process monitoring, fault diagnosis, nonlinear large-scale, sparse Bayesian, transfer learning, probabilistic relevance vector machine

## Abstract

Process monitoring plays an important role in ensuring the safety and stable operation of equipment in a large-scale process. This paper proposes a novel data-driven process monitoring framework, termed the ensemble adaptive sparse Bayesian transfer learning machine (EAdspB-TLM), for nonlinear fault diagnosis. The proposed framework has the following advantages: Firstly, the probabilistic relevance vector machine (PrRVM) under Bayesian framework is re-derived so that it can be used to forecast the plant operating conditions. Secondly, we extend the PrRVM method and assimilate transfer learning into the sparse Bayesian learning framework to provide it with the transferring ability. Thirdly, the source domain (SD) data are re-enabled to alleviate the issue of insufficient training data. Finally, the proposed EAdspB-TLM framework was effectively applied to monitor a real wastewater treatment process (WWTP) and a Tennessee Eastman chemical process (TECP). The results further demonstrate that the proposed method is feasible.

## 1. Introduction

Due to the increasing diversification of industrial demand, the combination of process and equipment results in system structures become increasingly complex. Therefore, if the operation status of a plant cannot be monitored comprehensively and efficiently, it will not only cause serious economic losses [1], but also may cause irreversible damage to social communities. Timely detection and prediction of faults has become a focus of attention in academia and industry [2,3,4]. Recently, data-driven process monitoring has developed as the best form of “whistleblower” for extreme or abnormal events in a plant. This is because the data-driven process monitoring method does not need to establish an accurate mechanism model; rather, it uses a data-driven model to establish a global monitoring method for complex large-scale industrial processes [5]. Moreover, data-driven monitoring methods have been successfully applied in many different scenarios [1,6,7,8,9,10,11]. Liu et al., proposed a variational Bayesian principal component analysis (PCA) model to effectively monitor a wastewater treatment process (WWTP) [6]. Ge et al., proposed a two-step information extraction strategy to monitor a Tennessee Eastman chemical process (TECP) [7]. Zhu et al., proposed a novel two-step strategy probabilistic independent component analysis- probabilistic PCA (PICA-PPCA) to improve the robustness of the traditional method [8]. However, the above-mentioned data-driven methods ignore some general characteristics. Firstly, due to the complexity of external disturbances, the collected datasets contain nonlinear information [12]. Therefore, the performance of the above-mentioned linear methods usually degrades. Secondly, due to the increasing complexity of industrial equipment, monitoring models require an increasing amount of real-time training data. When sufficient training data are not available to train a reliable model, the monitoring model produces false and missed alarms, resulting in unpredictable losses to the factory.

To address the nonlinear characteristics of industrial data, the academia and industrial communities have undertaken a significant amount of research [1,13,14]. Ma et al., proposed using a deep convolutional neural network to diagnose the faults of rotating machinery [1]. However, the neural network-based method has some disadvantages, such as high computational cost and poor interpretability, especially when the network layer increases, which leads to an increase in the required tuning parameters. Based on the above considerations, the kernel function method, as a powerful technology, has been effectively used to expand the traditional statistical monitoring model. Lee et al., used a kernel PCA (KPCA)-based method to monitor a nonlinear wastewater treatment system, and experiment results show that KPCA performed better than PCA [13]. Wang et al., proposed using kernel independent component analysis (KICA) to diagnose nonlinear process faults. The experimental results show that KICA is superior to ICA [14]. In addition, the kernel-based support vector machine (SVM) has become one of the most popular method [15]. Liu et al., presented an in-depth discussion on the application of kernel-based methods in industry [16].

However, the number of support vectors will increase rapidly as the size of training sets increases [17], thus unnecessarily increasing the computational burden. To overcome this problem, the computational burden can be reduced by increasing the sparsity of the model. Sparsity is an important and desirable property for algorithm design and model construction. Firstly, sparsity is able to control the complexity of the model and avoids over-fitting. Secondly, prediction using a sparse model is highly effective in computation. Inspired by [17], the RVM under the sparse Bayesian framework is selected accordingly. In recent years, RVM has gained more attention. Liu et al., used an RVM to predict the difficult-to-measure variables of a WWTP [18]. Wu et al., proposed a multi-kernel RVM to predict the quality-related faults of a WWTP [19]. Hu et al., developed an RVM to predict the remaining useful life (RUL) of field pump impellers [9]. Because the posterior distributions of many of the weights are sharply peaked around zero, the sparsity of the RVM can easily use automatic relevance determination (ARD) to remove the zero-weight “relevance” vectors [20]. Furthermore, the sparsity of the RVM can meet the needs of real-time monitoring of factories. Therefore, based on the sigmoid function, Bernoulli distribution, Bayesian derivation, and Markov chain rule, we re-derived the probability RVM, allowing the probabilistic relevance vector machine (PrRVM) to monitor WWTPs and the TE chemical process.

The re-derived PrRVM is still limited by the following factors. When the amount of training data is insufficient, the performance of the PrRVM is weakened. Based on previous research [21,22], one solution lies in augmentations of the available data. The training dataset can be artificially expanded through transformations of samples, such as adding additional noise to the raw data. Another approach is to create synthetic data to assist in model training [21]. However, these methods rely too heavily on the original data, and expansion of the dataset is subject to significant uncertainty, which can be counterproductive. To address the above problems, transfer learning is considered to be embedded in PrRVM. Transfer learning aims to transfer the learned knowledge from one domain (source domain) to another domain (target domain). Therefore, the insufficient training data problem can be potentially addressed by knowledge transformation from additional datasets with sufficient supervised information. Transfer learning can be divided into three categories [23]: instance-based transfer learning, feature-based transfer learning, and model-based transfer learning. Based on [24], we used adaptive boosting technology and instance-based transfer learning to update the weight vector of source domain (SD) data and labeled target domain (LTD) data. If SD data can improve the method performance, its weight is increased. For LTD data, when it is misclassified, its weight will be increased to ensure more attention is paid to its optimization in the next iteration. In each iteration period, the updated data will be used to train a novel PrRVM detection model.

Note that the data collected by the process industries (WWTP and TECP) are not designed for transfer learning. Therefore, the dataset must be split before executing the corresponding strategy. Firstly, the real-time collected TD dataset is split into two components: the first component is the labeled target domain (LTD) dataset, which is defined as the training dataset. The second component is the unlabeled target domain (ULTD) dataset, which is defined as the real-time testing dataset. The SD dataset is the auxiliary training dataset, which is the out-of-date dataset. Then, the SD dataset and LTD dataset are updated by adaptive boosting technology and transfer learning. To summarize, we propose a modified version of a PrRVM for fault diagnosis that can enable a high quantitative fault diagnosis performance in the design process. Additionally, transfer learning is embedded in the PrRVM to solve the problem of insufficient training data. The ensemble monitoring model constructed using two-layer iteration (weight iteration and hyperparameter iteration) with the ensemble rule is termed the ensemble adaptive sparse Bayesian transfer learning machine (EAdspB-TLM). Finally, key performance indicators (KPIs) are used to evaluate the performance of different methods.

The paper is organized as follows: Section 2 presents the basic theory of the approach. Section 3 provides a detailed formula derivation of the EAdspB-TLM. In Section 4, the EAdspB-TLM is used to monitor different types of faults, and the experiment results are discussed and analyzed. Finally, the paper ends with conclusions in Section 5.

## 2. Theoretical Foundation

### 2.1. Transfer Learning

The purpose of transfer learning is to gain knowledge from an environment (source domain) to help the learning task in a new environment (target domain) [23]. To facilitate the subsequent use of transfer learning algorithms, the general symbols related to transfer learning are defined as follows:

(1) Detection model Φ: X⟼Y, where X represents the training data or testing data. Y represents the corresponding sample label. In this study, the premise is to assume that the training data are not sufficient to train a reliable detection model Φ.
(2) Domain (D): The symbol of domain is represented by D={X,P(X)}, where $X=\left\{\begin{array}{ccc} x_{1} & \cdots & x_{n} \end{array}\right\}\in X$, X is a feature space. $D_{s}=\left\{X_{s},P\left(X_{s}\right)\right\}$ is the source domain (SD). $D_{t}=\left\{X_{t},P\left(X_{t}\right)\right\}=D_{t1}\cup^{\;}D_{t2}$ is the target domain (TD). $D_{t1}$ and $D_{t2}$ are the LTD and ULTD, respectively. In this paper, LTD data are used as the training data; ULTD data are used as the testing data.
(3) Task (T): T={Y,f(∗)}, Y∈{0,1} is the sample label. f(∗) is the corresponding prediction function, f(X)=P(Y|X). Its task is to minimize the deviation between the predicted label and the real label Y.

### 2.2. Sparse Bayesian for Fault Diagnosis

The essence of data-driven fault diagnosis is to identify the running state of the equipment. The corresponding labels can be set for different running states; for example, the data label of the normal working condition is set to 0, and the data label of the fault state is set to 1. Then the fault diagnosis model in the framework of a sparse Bayesian (PrRVM) is equivalent to a supervised classifier. In this study, the PrRVM is a sparse model with probabilistic output. Suppose the training dataset is ${\left\{x_{i},y_{i}\right\}}_{i=1}^{n}$, where $x_{i}$. is the put data, then $y_{i}\in \left\{0,1\right\}$. is the corresponding label. The prediction formula of PrRVM can be expressed as follows:

(1) $$y_{j}=\Phi \left(x_{j};w\right)=\overset{n}{\underset{i=0}{\sum }}w_{i}f_{i}\left(x_{j}\right)+\varepsilon$$

where $w=\left[w_{0},w_{1},\cdots ,w_{n}\right]$ is the weight vector. ε represents the additive noise, let $\varepsilon \sim N\left(0,\sigma^{2}\right)$. $f_{i}\left(x_{j}\right)=k\left(x_{j},x_{i}\right)$ is kernel function, which aims to map low dimensional non-separable data to high dimensional space. When the weight vector w and variance $\sigma^{2}$ are known, the label vector $y={\left[y_{1},w_{2},\cdots ,w_{n}\right]}^{T}$ can be derived using the following probability expression:

(2) $$p\left(y|w,\sigma^{2}\right)=\overset{n}{\underset{i=1}{\prod }}N\left(y_{i}|u,\sigma^{2}\right)={\left(2\pi \sigma^{2}\right)}^{-n/2}\exp\left(\frac{-{\left\|y-\Psi w\right\|}^{2}}{2\sigma^{2}}\right)$$

according to [17], Ψ is the n×(n+1) “design” matrix, where

(3) $$\Psi =\left[\begin{array}{ccc} \begin{array}{cc} 1 & k\left(x_{1},x_{1}\right)\\ 1 & k\left(x_{2},x_{1}\right) \end{array} & \cdots & \begin{array}{c} k\left(x_{1},x_{n}\right)\\ k\left(x_{2},x_{n}\right) \end{array}\\ \vdots & \ddots & \vdots \\ \begin{array}{cc} 1 & k\left(x_{n},x_{1}\right) \end{array} & \cdots & k\left(x_{n},x_{n}\right) \end{array}\right]$$

w and $\sigma^{2}$ can be estimated by expectation maximization, but it is subject to over-fitting [17]. To avoid over-fitting, a common approach is to impose some additional constraints on the parameters. We use Bayesian strategy and define an explicit prior probability distribution on the parameters to “constrain” the parameters. Assuming that the zero-mean Gaussian prior distribution on the weight vector w can be expressed as follows:

(4) $$p\left(w|a\right)=\overset{n}{\underset{i=0}{\prod }}N\left(w_{i}|0,a_{i}^{-1}\right)$$

$a=\left[a_{0},a_{1},\cdots ,a_{n}\right]$ is the hyperparameter vector. w and $\sigma^{2}$ can be further solved by Bayesian inference and rules. Here, we first assume that w and $\sigma^{2}$ are known, and then derive the solution formula of the classification problem. Firstly, the logistic sigmoid function $\sigma \left(z\right)={\left(1+e^{-z}\right)}^{-1}$ is introduced. Assuming that the data obey the Bernoulli distribution, the corresponding likelihood function can be expressed as follows:

(5) $$p\left(y|w,\sigma^{2}\right)=\overset{n}{\underset{i=1}{\prod }}\sigma {\left(\Phi \left(x_{i};w\right)\right)}^{y_{i}}{\left[1-\sigma \left(\Phi \left(x_{i};w\right)\right)\right]}^{1-y_{i}}$$

When the predicted value y=1, it indicates that the system is out of control.

## 3. Ensemble Adaptive Sparse Bayesian Transfer Learning Machine for Process Monitoring

### 3.1. Adaptive Boosting Technology and Transfer Learning

**Figure 1:**
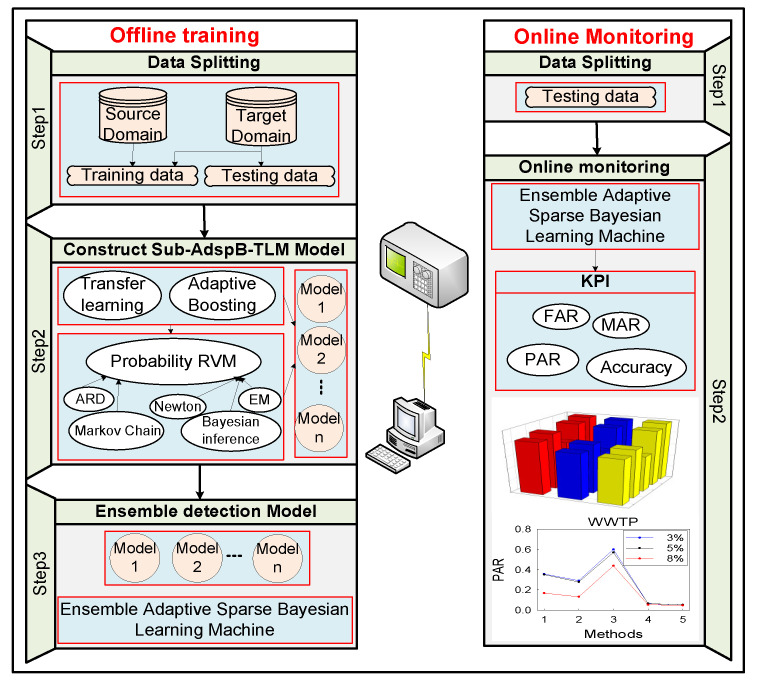
Schematic diagram of the proposed process monitoring framework.

The proposed process monitoring framework is shown in Figure 1. The adaptive sparse Bayesian transfer learning machine is mainly composed of two components. The first component is the adaptive boosting technology in the transfer learning framework, and the second component is the PrRVM fault diagnosis model in the Bayesian framework. The first part was proposed by Dai et al. [24], and named the TrAdaBoost algorithm. In this paper, TrAdaBoost is used to assign the data weights. Before the algorithm is implemented, suppose that the following symbols represent the divided SD data and label: Data: $X_{s}\in R^{p_{s}\times n_{s}}$, label $Y_{s1}\in R^{1\times n_{s}}$. LTD data: $X_{t_{1}}\in R^{p_{t}\times n_{t1}}$, and the corresponding label $Y_{t_{1}}\in R^{1\times n_{t1}}$. ULTD data: $X_{t_{2}}\in R^{p_{t}\times n_{t2}}$. $n_{s}$ and $p_{s}$ represent the source domain sample number and the corresponding monitored variable number, respectively. $n_{t1}$ and $n_{t1}$ represent the number of samples of LTD and ULTD, respectively. $p_{t}$ is the number of monitored variables in the target domain, and $p_{t}=p_{s}$. The procedure of the TrAdaBoost algorithm can be derived as follows:

Firstly, initialize the weight vector $\tau^{1}=\left(\begin{array}{ccc} {\tau_{1}}^{1} & \cdots & {\tau_{n_{s}+n_{t1}}}^{1} \end{array}\right)$, where:

(6) $$\tau_{i}{}^{1}=\{\begin{array}{c} \frac{1}{n_{s}}i=1,\cdots ,n_{s}\\ \frac{1}{n_{t1}}i=n_{s}+1,\cdots ,n_{s}+n_{t1} \end{array}$$

Secondly, call the detection model (PrRVM); according to the detection results, the corresponding data weights are updated process as follows:

Based on [24], set $\beta =1/\left(1+\sqrt{2ln\frac{n_{s}}{L}}\right)$. L is the number of iterations. Re-define the weight of SD data and LTD data, where:

(7) $$\tau_{i}^{j}=\frac{\tau_{i}{}^{j}}{\sum_{i=1}^{n_{s}+n_{t1}}\tau_{i}{}^{j}}$$

The sub-PrRVM ($\varphi_{j}$) is trained using the data with the weight distribution of Equation (7). Then, return the detection model $\varphi_{j}$: X⟼Y. Calculate the error of $\varphi_{j}$ on $X_{t_{1}}$:

(8) $$\epsilon_{j}=\overset{n_{s}+n_{t1}}{\underset{i=n_{s}+1}{\sum }}\frac{\omega_{i}^{j}\left|\varphi_{j}\left(x_{i}\right)-Y_{i}\right|}{\sum_{i=n_{s}+1}^{n_{s}+n_{t1}}\tau_{i}^{j}}$$

The change parameter $\beta_{j}$ of $X_{t_{1}}$ is obtained as follows:

(9) $$\beta_{j}=\frac{\epsilon_{j}}{1-\epsilon_{j}}$$

Then, updating the weight vector:

(10) $$\tau_{i}{}^{j+1}=\{\begin{array}{c} {\tau_{i}}^{j}\beta^{\left|\phi_{j}\left(x_{i}\right)-Y_{i}\right|}i=1,\cdots ,n_{s}\\ {\tau_{i}}^{j}\beta_{j}{}^{-\left|\phi_{j}\left(x_{i}\right)-Y_{i}\right|}i=n_{s}+1,\cdots ,n_{s}+n_{t1} \end{array}$$

Finally, L sub-detection models ($\varphi_{1}^{*},\varphi_{2}^{*},\cdots ,\varphi_{L}^{*}$) are obtained through L iterations of the whole process.

In this paper, the common formulas are presented. The corresponding rigorous theoretical proof can is provided in previous research papers. For example, the weight distribution formula refers to the *Hedge* (*β*) theorem [25]. The proof of $1/(1+\sqrt{2ln\frac{n_{s}}{L}}$ can be found in [26].

### 3.2. Adaptive Probabilistic Relevance Vector Machine

In this section, the evolution steps of the adaptive PrRVM within the joint framework of transfer learning and sparse Bayesian are further deduced. According to Section 2.2, we can obtain the probability derivation process of $p\left(y|w,\sigma^{2}\right)$. In the derivation process, w and $\sigma^{2}$ need to be updated in each training process. Therefore, the iterative process of w and $\sigma^{2}$ in the transfer learning framework is re-defined. Assume that the posterior probability of $w,\sigma^{2}$, and a can be expressed as $p\left(w,\sigma^{2},a|y\right)$. According to Bayesian inference, $p\left(w,\sigma^{2},a|y\right)$ can be further decomposed as follows:

(11) $$p\left(w,\sigma^{2},a|y\right)=\frac{p\left(w,y,\sigma^{2},a\right)}{p\left(y,\sigma^{2},a\right)}*\frac{p\left(y,\sigma^{2},a\right)}{p\left(y\right)}=p\left(w|y,\sigma^{2},a\right)p\left(\sigma^{2},a|y\right)$$

The solution of unknown parameters $w,\sigma^{2}$, and a depends on $p\left(w|y,\sigma^{2},a\right)$ and $p\left(\sigma^{2},a|y\right)$. For the classification problem, the posterior probability of weight w cannot be calculated directly. Here, we assume that the hyperparameter vector a is known, and $p\left(w|y,\sigma^{2},a\right)$ can be further derived as follows:

(12) $$p\left(w|y,\sigma^{2},a\right)=\frac{p\left(w,y,\sigma^{2},a\right)}{p\left(y,\sigma^{2},a\right)}=\frac{p\left(y|w,\sigma^{2},a\right)p\left(w|\sigma^{2},a\right)p\left(\sigma^{2},a\right)}{p\left(y|\sigma^{2},a\right)p\left(\sigma^{2},a\right)}$$

To facilitate the subsequent derivation, we omit the indirect relationship between variables. According to the Bayes rule and Markov property, Equation (12) can be further transformed as follows:

(13) $$p\left(w|y,\sigma^{2},a\right)=\frac{p\left(y|w,\sigma^{2}\right)p\left(w|a\right)}{p\left(y|\sigma^{2},a\right)}=\frac{p\left(y|w,\sigma^{2}\right)p\left(w|a\right)}{\int^{\;}p\left(y|w,\sigma^{2}\right)p\left(w|a\right)dw}$$

Therefore, $p\left(w|y,\sigma^{2},a\right)\propto p\left(y|w,\sigma^{2}\right)p\left(w|a\right)$. In addition, we can further deduce $p\left(\sigma^{2},a|y\right)\propto p\left(y|\sigma^{2},a\right)p\left(\sigma^{2}\right)p\left(a\right)$. Because we cannot directly solve $p\left(w|y,\sigma^{2},a\right)$ and $p\left(\sigma^{2},a|y\right)$, we can solve $p\left(y|\sigma^{2},a\right)$ and $p\left(y|w,\sigma^{2}\right)$ to derive the desired result. Here, the formulas of $p\left(y|w,\sigma^{2}\right)$ and p(w|a) can be expressed as follows:

(14) $$p\left(y|w,\sigma^{2}\right)=\overset{n}{\underset{i=1}{\prod }}\sigma {\left(\Phi_{i}\right)}^{y_{i}}{\left[1-\sigma \left(\Phi_{i}\right)\right]}^{1-y_{i}}$$

(15) $$\begin{array}{c} p\left(w|a\right)=\overset{n}{\underset{i=0}{\prod }}N\left(w_{i}|0,a_{i}^{-1}\right)=\overset{n}{\underset{i=0}{\prod }}{\left(2\pi a_{i}^{-1}\right)}^{-\frac{1}{2}}\exp(-\frac{1}{2}a_{i}w^{2})\\ ={\left(2\pi \right)}^{-\frac{n+1}{2}}{\left|\Lambda \right|}^{\frac{1}{2}}\exp\left(-\frac{1}{2}w^{T}\Lambda w\right) \end{array}$$

where $\Phi_{i}=\Phi \left(x_{i};w\right)$, Λ is the diagonal matrix:

(16) $$\Lambda =\left(\begin{array}{cccc} a_{0} & 0 & \cdots & 0\\ 0 & a_{1} & \cdots & 0\\ \vdots & \vdots & \ddots & 0\\ 0 & 0 & \cdots & a_{n} \end{array}\right)$$

When the hyperparameter vector a is fixed, Newton’s method can be used to solve $p\left(y|w,\sigma^{2}\right)p\left(w|a\right)$:

(17) $$\begin{array}{c} \log\left(p\left(y|w,\sigma^{2}\right)p\left(w|a\right)\right)=\log p\left(y|w,\sigma^{2}\right)+\log p\left(w|a\right)\\ =\overset{n}{\underset{i=1}{\sum }}[y_{i}\log\sigma \left(\Phi_{i}\right)+\left(1-y_{i}\right)*\log\left(1-\sigma \left(\Phi_{i}\right)\right]+\Theta -\frac{1}{2}w^{T}\Lambda w \end{array}$$

where $\Theta =\log\left[{\left(2\pi \right)}^{-\frac{n+1}{2}}\prod_{i=0}^{n}a_{i}^{\frac{1}{2}}\right]$, because Equation (17) is a penalized logistic log-likelihood function, and necessitates iterative maximization [17]. The second-order Newton method is used to derive the target function. In addition, it can be further deduced that $\log p\left(w|y,\sigma^{2},a\right)\propto log\left(p\left(y|w,\sigma^{2}\right)p\left(w|a\right)\right)$. Based on [27], we should take the derivative of w. Assuming that the solved extreme point is $w_{MP}$, the second derivative result of w can be expressed as follows:

(18) $$\begin{array}{l} G=\frac{\partial^{2}}{\partial w}\left(\log p\left(w|y,\sigma^{2},a\right)\right){|}_{w_{MP}}\\ \;\;\;\;\;\;=\frac{\partial^{2}}{\partial w}\left(\overset{n}{\underset{i=1}{\sum }}[y_{i}\log\sigma \left(\Phi_{i}\right)+\left(1-y_{i}\right)*\log\left(1-\sigma \left(\Phi_{i}\right)\right]+\Theta -\frac{1}{2}w^{T}\Lambda w\right)\\ \;\;\;\;\;\;=-\Psi^{T}Η\Psi -\Lambda \end{array}$$

where Ψ is shown in Equation (3). Η is the diagonal matrix, $Η=\mathrm{diag}\left(h_{1},h_{2},\cdots h_{n}\right)$, and $h_{i}=\sigma \left(\Phi_{i}\right)(1-\sigma \left(\Phi_{i}\right)$. Η can be written as

(19) $$Η=\left(\begin{array}{ccc} \sigma \left(\Phi_{1}\right)(1-\sigma \left(\Phi_{1}\right) & \cdots & 0\\ \vdots & \ddots & \vdots \\ 0 & \cdots & \sigma \left(\Phi_{n}\right)(1-\sigma \left(\Phi_{n}\right) \end{array}\right)$$

Based on [28], the covariance matrix $\sum^{\;}$ and $\left(-\Psi^{T}Η\Psi -\Lambda \right)$ can be linked as follows:

(20) $$\Sigma ={\left(-G\right)}^{-1}={\left(\Psi^{T}Η\Psi +\Lambda \right)}^{-1}$$

It can be seen that the Laplace approximation effectively maps the classification problem to a regression problem with data-dependent noise [29], with the inverse noise variance for ε given by $\sigma \left(\Phi_{i}\right)(1-\sigma \left(\Phi_{i}\right)$. In addition, according to $\frac{\partial }{\partial w}\left(\log p\left(w|y,\sigma^{2},a\right)\right){|}_{w_{MP}}=0$ and Σ, $w_{MP}$ can be further derived as follows:

(21) $$w_{MP}=\Sigma \Psi^{T}Ηy$$

Next, we can iteratively update hyperparameter vector a by fixing Σ and $w_{MP}$. According to the relation $p\left(\sigma^{2},a|y\right)\propto p\left(y|\sigma^{2},a\right)p\left(\sigma^{2}\right)p\left(a\right)$, we only need to further simplify $\log(p\left(y|\sigma^{2},a\right)$), and then repeat the previous derivation steps. The following relation can be obtained:

(22) $$\frac{\partial }{\partial a_{i}}\left(\log p\left(y|\sigma^{2},a\right)\right)=\frac{1}{2a_{i}}-\frac{1}{2}{\sum^{\;}}_{ii}-\frac{1}{2}u_{i}^{2}=0$$

where $u_{i}=\Sigma \Psi^{T}Ηy=w_{MP}$. Equation (22) can be further converted as follows:

(23) $$\alpha_{i}^{new}=\frac{1-\alpha_{i}*\Sigma_{ii}}{{\left(u_{i}\right)}^{2}}$$

### 3.3. Updating the Weight Vector and Sparse Analysis

In this section, the weight w is defined as the “hidden” variable. Then a general algorithm of expectation maximization (EM) is selected accordingly. EM mainly includes an expectation (E) step and a maximization (M) step. The adaptive PrRVM derived in this paper is used for classification. Assuming that ε is random additive noise, when the output is $\sum_{i=0}^{n}w_{i}f_{i}\left(x_{j}\right)+\varepsilon \geq 0$, the corresponding prediction label is $p\left(y=1|w,\sigma^{2}\right)=\prod_{i=1}^{n}\sigma {\left(\Phi_{i}\right)}^{y_{i}}{\left[1-\sigma \left(\Phi_{i}\right)\right]}^{1-y_{i}}$. The probit mode can be presented as follows:

(24) $$p\left(y=1|w,\sigma^{2}\right)=p\left(\overset{n}{\underset{i=0}{\sum }}w_{i}f_{i}\left(x_{j}\right)+\varepsilon \right)\geq 0)$$

The probability derivation of the weight vector w can be expressed as $p\left(w|y,\sigma^{2},a\right)\propto p\left(y|w,\sigma^{2}\right)p\left(w|a\right)$. The corresponding log-posterior is $\log\left(p\left(w|y,\sigma^{2},a\right)\right)=\log p\left(y|w,\sigma^{2}\right)+\log p\left(w|a\right)$. Suppose that the hyperparameter at time t is denoted as $\alpha_{i}^{\left(t\right)}$. According to [30], define a new Q function and let $Q(w^{t}|w^{t+1})=\log\left(p\left(w^{t}|y^{\left(t+1\right)},{\left(\sigma^{2}\right)}^{\left(t\right)},a^{\left(t\right)}\right)\right)$. The following ***expectation step*** can be derived:

(25) $$Q\left(w^{t}|w^{t-1}\right)=\log p\left(y^{\left(t\right)}|w^{t},{\left(\sigma^{2}\right)}^{\left(t\right)}\right)+\log p\left(w^{t}|a^{\left(t\right)}\right)$$

In the ***maximization step*** stage, we can update $a^{\left(t+1\right)}$ at the time of t+1 through $w^{t}$. Calculating the partial derivative of Q(∗), we can then obtain the following $\alpha_{i}^{\left(t+1\right)}:$

(26) $$\alpha_{i}^{\left(t+1\right)}=\frac{1}{\Sigma_{ii}{}^{t}+{\left({u_{i}}^{t}\right)}^{2}}$$

This scenario illustrates that the hyperparameters can be updated adaptively with available new inputs. In addition, during the update process, it is found that some $\alpha_{i}^{new}$ will approach infinity. At this time, the automatic correlation decision (ARD) can be used to update the corresponding u and Σ. When $\alpha_{i}^{new}$ approaches infinity, ARD will make the corresponding $u_{i}$ and $\Sigma_{ii}$ equal to zero [20]. $w_{i}$ is updated to zero. In this way, the matrix becomes sparse. Finally, it is assumed that the parameter probability estimation of the adaptive PrRVM is expressed by the following symbols: the weight parameter $w^{*}=\left[w_{0}^{*},w_{1}^{*},\cdots ,w_{n}^{*}\right]$ and $\Lambda^{*}=diag\left\{a_{0}^{*},a_{1}^{*},\cdots ,a_{n}^{*}\right\}$. Additionally, $\mathrm{rank}\left(\Lambda^{*}\right)<n+1$. The iteratively updated sparse matrix $\Sigma^{*}=\left[\begin{array}{cc} 0 & 0\\ 0 & {\left(\Psi^{*T}{Η}^{*}\Psi^{*}+\Lambda^{*}\right)}^{-1} \end{array}\right]$, and the final prediction label $y^{*}$ can be obtained.

### 3.4. Ensemble Detection Model and Key Performance Indicator

The finite number of adaptive sparse Bayesian transfer learning machines $\left\{\varphi_{1}^{*},\varphi_{2}^{*},\cdots ,\varphi_{L}^{*}\right\}$ can be derived by Section 3.1 and Section 3.3. Moreover, the effective system decision making needs to consider the detection results of multiple adaptive sparse Bayesian transfer learning machines simultaneously. Based on [24], the following ensemble detection model can be constructed:

(27) $$\varphi^{*}\left(x\right)=\{\begin{array}{c} 1,\sum_{j=\left[\frac{L}{2}\right]}^{L}ln\left(\frac{1}{\beta_{j}}\right)\varphi_{j}^{*}\left(x\right)\geq \frac{1}{2}\sum_{j=\left[\frac{L}{2}\right]}^{L}ln\left(\frac{1}{\beta_{j}}\right)\\ 0,Otherwise \end{array}$$

where:

$$\beta_{j}=\epsilon_{j}/\left(1-\epsilon_{j}\right),i.e.,\beta_{j}=\sum_{i=n_{s}+1}^{n_{s}+n_{t1}}\frac{\omega_{i}^{j}\left|\phi_{j}^{*}\left(x_{i}\right)-Y_{i}\right|}{\sum_{i=n_{s}+1}^{n_{s}+n_{t1}}\omega_{i}^{j}}/(1-\sum_{i=n_{s}+1}^{n_{s}+n_{t1}}\frac{\omega_{i}^{j}\left|\phi_{j}^{*}\left(x_{i}\right)-Y_{i}\right|}{\sum_{i=n_{s}+1}^{n_{s}+n_{t1}}\omega_{i}^{j}})$$

When the above ensemble detection model is obtained, it is necessary to verify the performance of the model. KPIs are the critical decision tools for evaluating the method performance. They are the quantifiable and results-based statements. In this study, missed alarm rate (MAR), false alarm rate (FAR), accuracy, and pre-alarm rate (PAR) were carefully selected as KPIs. The corresponding formulas are as follows:

(28) $$\mathrm{MAR}=\mathrm{Fr}\left(\mathrm{Normal}|\mathrm{Fault}\right)=\frac{FN}{FN+TP}$$

(29) $$\mathrm{FAR}=\mathrm{Fr}\left(\mathrm{Fault}|\mathrm{Normal}\right)=\frac{FP}{FP+TN}$$

(30) PAR=ϖMAR+(1−ϖ)FAR

(31) $$\mathrm{accuracy}=\frac{TP+TN}{TP+FP+TN+FN}$$

Note that “normal” is the fault-free condition. Fr(|) represents the conditional frequency [12]. TP is true positive; TN represents true negative; FP is false positive. The PAR is constructed by combining the false alarm and missed alarm indicators [12]. ϖ is the weight parameter (0≤ϖ≤1).

## 4. Case Studies

### 4.1. Experimental Design and Compared Approaches

**Figure 2:**
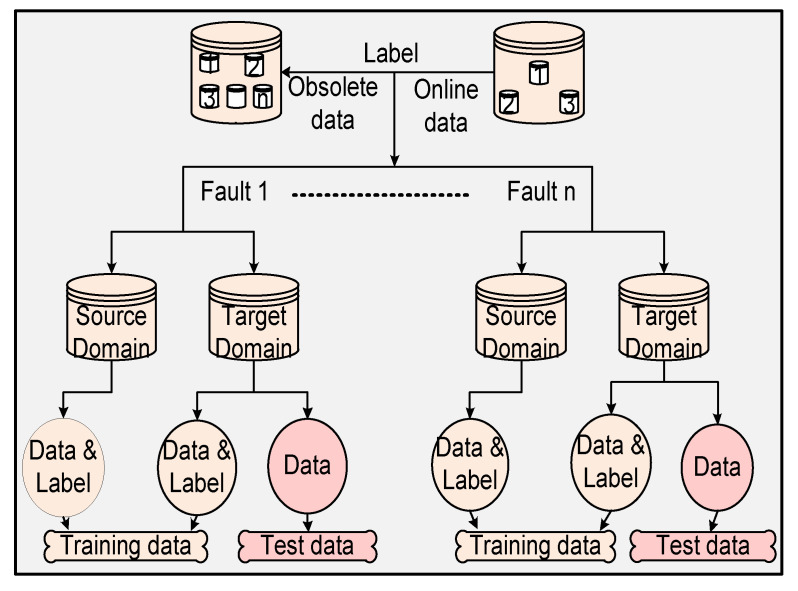
The flowchart of data splitting.

In this section, the dataset splitting steps are introduced in detail. Firstly, the SD data are filtered according to LTD data, in such a way that SD data and LTD data have the same types of labels. The data splitting is shown in Figure 2. Firstly, LTD data have the same distribution as ULTD data. IN contrast to the previous transfer learning, we combine the SD data and LTD data to form a new training set, and use the ULTD data as a testing set. The proportion of LTD data is 1%–10%. The “proportion” formula is defined as PR = $\frac{\left\|\mathrm{LTD}\right\|}{\left\|\mathrm{SD}\right\|}$, where ‖LTD‖ is the number of labeled samples in the target domain, and ‖SD‖ is the number of samples in the source domain. Moreover, the main aim of the experiment is to monitor the single fault of the system. Multiple fault cases can be expanded accordingly. To verify the proposed method, traditional statistical methods and transfer learning methods were used to monitor a chemical plant and WWTP simultaneously. The traditional statistical methods PCA-T^2^ [31], SVM [32], and RVM [17] were trained using LTD data. RVMt and the proposed method were trained by the SD data and LTD data simultaneously.

In this study, the proposed EAdspB-TLM framework was used to monitor the TE chemical plant and a full-scale wastewater treatment plant (WWTP). The main tools used in the study were a personal computer (PC), MATLAB R2016a, SigmaPlot 12 and office software. The parameters of the PC are CPU Intel Core i7-6700HQ, 8 GB RAM, and 1 TB SSD. The data are from a TE simulation platform and a real WWTP.

### 4.2. Case Study on the Tennessee Eastman Chemical Process

#### 4.2.1. Background

**Figure 3:**
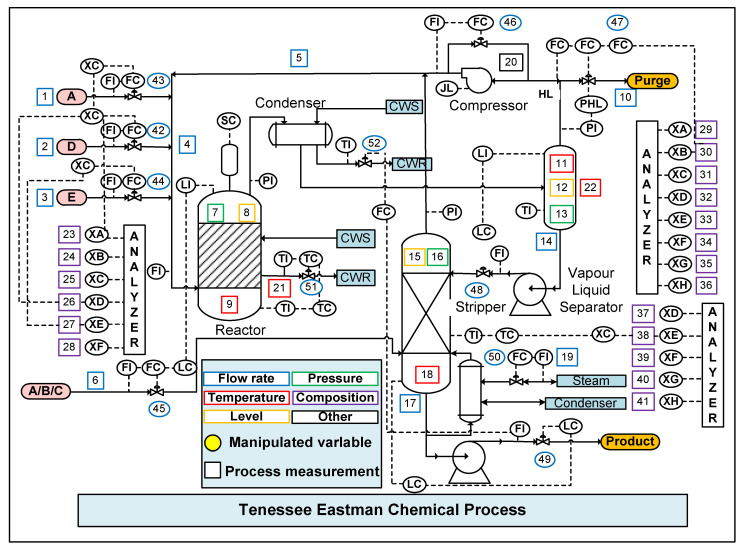
Schematic layout of the Tennessee Eastman chemical process (TECP) [33].

The Tennessee Eastman chemical process (TECP) was designed by a chemical company as a testing process control and diagnosis method. As shown in Figure 3, the TECP consists of five core units: reactor, compressor, stripper, condenser, and separator. The process includes measured variables and manipulated variables. There are four gaseous reactants (A, B, C, D, E) and two liquid products (G and H). The reaction equation is as follows:

(32) $$\{\begin{array}{c} A\left(g\right)+C\left(g\right)+D\left(g\right)\to G\left(\mathrm{liq}\right)\\ A\left(g\right)+C\left(g\right)+E\left(g\right)\to H\left(\mathrm{liq}\right)\\ A\left(g\right)+E\left(g\right)\to F\left(\mathrm{byproduct}\right)\\ 3D\left(g\right)\to 2F\left(\mathrm{byproduct}\right) \end{array}$$

where F is the byproduct in the reactor, and the process is irreversible. More detailed reaction information of the TEP can be found in [33]. Moreover, the simulation program and operation introduction can be downloaded from http://depts.washington.edu/control/LARRY/TE/download.html#Basic_TE_Code. According to [33], 52 observation variables were selected for process monitoring depending on the process importance. Firstly, the platform started with a 25 h steady state. Then, the simulation ran for 97 h in each case. The sampling time was set up as 3 min. The source domain dataset resulted from the initial 59 h simulation. The corresponding dataset started with a normal working condition, but with faults being imposed after 39 simulation hours. Target domain (TD) data were collected from the simulation period of 59–97 h. TD data mainly includes two parts: the LTD and ULTD datasets. In this study, the ULTD dataset is defined as the testing dataset.

#### 4.2.2. Analysis and Discussion of Experimental Results

EAdspB-TLM differs from previous modeling methods. It has the abilities of adaptive adjustment and transfer learning. To verify the performance of the proposed EAdspB-TLM, five fault cases were used. The fault type description is shown in Table 1. Simultaneously, according to the engineering experience and cross-validation, the proposed method basic parameters were set by trial and error: the kernel function is “Gauss” ϖ = 0.6, and the maximum number of iterations and period are 1000 and 100, respectively.

In this study, step and sticking faults are the most noteworthy among the above five type faults. When the external disturbance is strong, it is easy to cause step faults of the sensor or other equipment. Table 2 presents monitoring results for Fault 1. In addition to the transfer learning methods of EAdspB-TLM, unsupervised statistical (PCA) and supervised statistical (SVM, RVM) methods are also presented. It is worth noting that the step fault of D feed temperature is a kind of fault that is difficult to monitor. Because the abnormality is not obvious, most monitoring models cannot effectively monitor this fault [3,34]. According to the experimental results, when PR = 8%, the detection accuracy of EAdspB-TLM is the highest among the five methods, with an accuracy of 87.2%. When PR = 3%, the accuracy of EAdspB-TLM is only 85.48%. In addition, the PAR of EAdspB-TLM is the lowest among the five methods. For example, when PR = 8%, the PAR of EAdspB-TLM is 13.52%. Moreover, the PAR of PCA-T^2^ and RVM are 49.06% and 16.83%, respectively. Moreover, the missed alarm rate of EAdspB-TLM is the lowest among the five methods. This shows that the proposed EAdspB-TLM is effective.

Fault 5 relates to a control problem with the reactor cooling water valve, which is a common sticking fault in the Tennessee Eastman chemical process (TECP). The reactor is an important component in the normal operation of the chemical plant. Once the fault occurs, other components (reactor, compressor, etc.) will not function normally. Therefore, it is imperative to monitor Fault 5 in real-time. The monitoring results for Fault 5 are tabulated in Table 3. When the PR value increased from 3% to 8%, the detection accuracy of EAdspB-TLM improved from 93.22% to 96.88%. Moreover, the detection accuracy of EAdspB-TLM is much higher than that of the other four methods. It is worth noting that when the PR value reaches 8%, the accuracy of EAdspB-TLM is 96.88%. The detection accuracy of EAdspB-TLM to Fault 5 is much higher than that of Fault 1. This indicates that the complexity of Fault 1 is higher than that of Fault 5.

**Figure 4:**
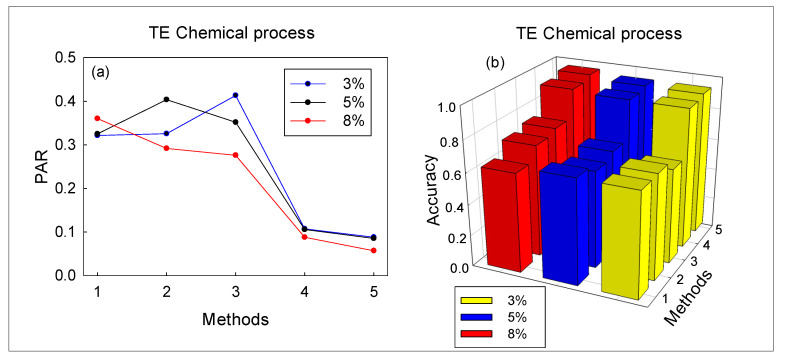
Average of comprehensive key performance indicators (KPIs) based on the proposed method and four other methods: (**a**) pre-alarm rate (PAR); (**b**) accuracy.

Overall, the performance of the monitoring method improves with the increase of PR value. Table 4 shows the average values of false and missed alarms of the five methods, that is, the average value of all faults in monitoring the TE chemical process. In terms of false alarm, when PR increases from 3% to 8%, false alarms of EAdspB-TLM decrease from 2.2% to 1.62%, false alarms of RVM decrease from 21.6% to 5.12%, and false alarms of SVM decrease from 32.02% to 19.06%. This shows that with the increase of PR, the false alarms of the methods will gradually decrease. In addition, the average value of the two comprehensive KPIs is shown in Figure 4: the PR alarm rate is shown in Figure 4a, and the fault diagnosis accuracy is shown in Figure 4b. It can be seen that with the increase of PR value, the fault diagnosis accuracy of the five monitoring methods gradually increases. In addition, the EAdspB-TLM method has the highest fault diagnosis accuracy; when the PR value increases to 8%, the accuracy of EAdspB-TLM is 94.61%.

### 4.3. Case Study of Full-Scale WWTP

#### 4.3.1. Background

**Figure 5:**
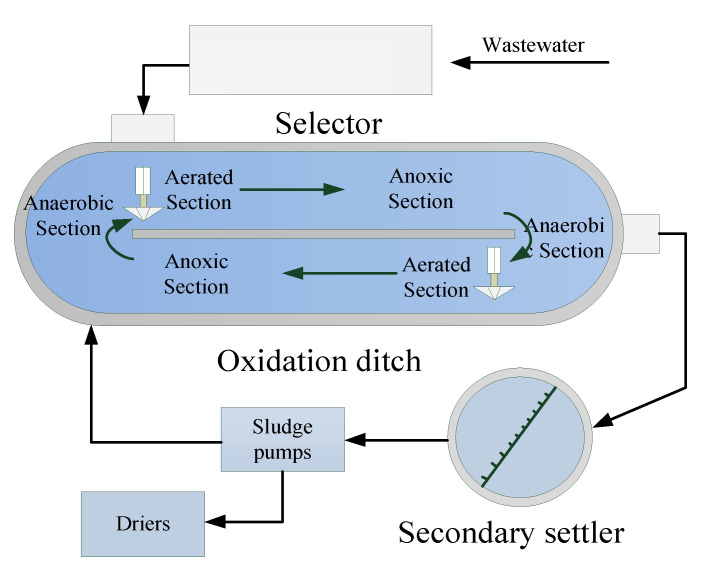
Schematic layout of a real full-scale wastewater treatment process (WWTP).

In this case study, the proposed method was used to monitor a real full-scale WWTP. The plant serves a population of 480,000, with a daily treatment flow of 170,000 m^3^ and a hydraulic retention time of 16.5 h. A long solid residence time (SRT) is used to achieve good nitrogen removal performance, and is typically maintained at 15–22 days. The schematic of the WWTP is shown in Figure 5. It is mainly composed of three components: selector, oxidation ditch, and secondary settler. Due to external disturbances, such as weather, temperature, and sludge activity, the filamentous sludge bulking occurs frequently and is difficult to monitor online in real time. The data were collected from 1 September to the following 31 March. Fifteen observation variables were selected as modeling variables. The sampling interval was one day and filamentous sludge bulking occurred during this period. The source domain dataset is based on the first samples of 110 days. This dataset starts with normal working conditions, but with faults occurring after 70 sample days.

#### 4.3.2. Analysis and Discussion of Experimental Results

**Figure 6:**
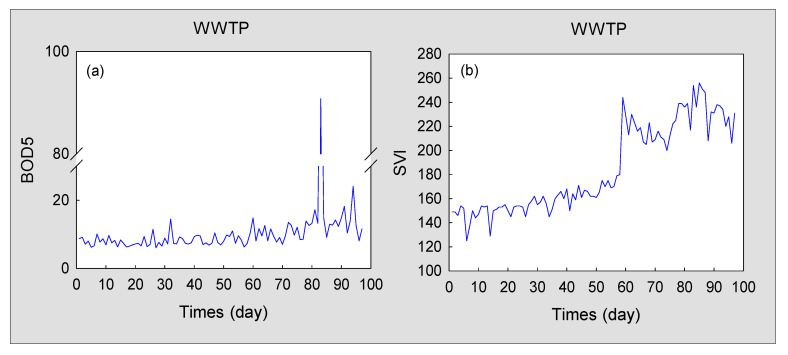
Isolated variables change curves of sludge bulking: (**a**) Five-day biochemical oxygen demand (BOD_5_); (**b**) the sludge volume index (SVI).

Filamentous sludge bulking is a type of drift fault [35]. In contrast to the abrupt fault, sludge bulking may return to normal after the self-regulation of microorganisms in the early stage [36]. During this period, the abnormality is less obvious (Figure 6). Figure 6a shows the dynamic trend of BOD_5_ (the five-day biochemical oxygen demand), and Figure 6b shows the curve of the sludge volume index (SVI). These can be used to determine if sludge bulking occurs in the WWTP. Although these indicators can be used to identify whether there is sludge bulking in the WWTP, the experiment is time-consuming. Therefore, real-time monitoring of the WWTP cannot be effectively implemented. In addition, the consecutive filamentous sludge bulking will cause the secondary pollution to the environment. Therefore, it is desirable to design an effective method for real-time monitoring of sludge bulking of WWTPs.

**Figure 7:**
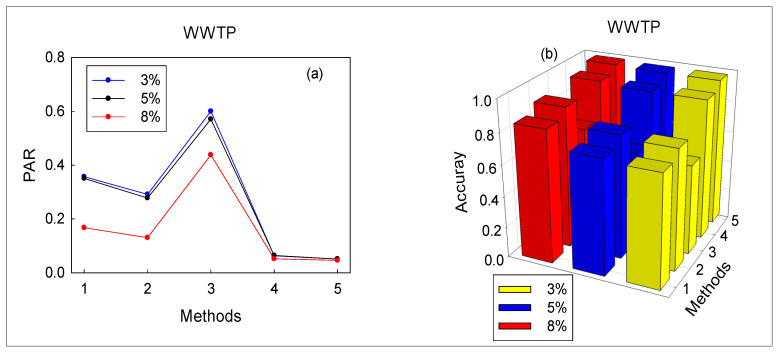
Average of comprehensive KPIs based on the proposed method and four other methods: (**a**) PAR; (**b**) accuracy.

When the data are obtained by the TECP, the technology of Section 4.1 was used to split the data accordingly. Then the proposed method and the other four methods were simultaneously used to monitor the wastewater treatment process; the false alarm rate, missed alarm rate, accuracy, and pre-alarm-rate of the five methods are tabulated in Table 5. Because sludge bulking is a slow drift fault, false and missed alarms become more obvious. When PR = 8%, the false alarm rate of PCA-T^2^ is 11.11%. The FAR of the SVM and RVM are both 1.85%. In comparison, the false alarm rate of EAdspB-TLM is zero. Furthermore, EAdspB-TLM is not the best in terms of missed alarms. The missed alarm rate of RVMt is higher than that of EAdspB-TLM. This unconventional result implies that EAdspB-TLM may not always be optimal. Thus, we need to further explore the effectiveness of EAdspB-TLM using the comprehensive KPIs (PAR and accuracy). According to Table 5 and Figure 7, the EAdspB-TLM-based pre-alarm rate is the lowest among the five methods (Figure 7a). For example, when PR = 8%, the PAR of EAdspB-TLM is 4.62%. In addition, the PARs of the comparison methods RVMt and SVM are 5.19% and 13.05%, respectively. In addition, when the PR value increased from 3% to 8%, the pre-alarm rate of EAdspB-TLM decreased from 5.09% to 4.62%. Based on the above analysis, we can conclude that the performance of EAdspB-TLM is the best among the six monitoring methods. At the same time, with the increase of PR value, the performance of the six methods is improved. Additionally, the fault diagnosis accuracy further verifies this conclusion, which is shown in Figure 7b. When PR = 8%, the average detection accuracy of EAdspB-TLM reaches 96.77%.

## 5. Conclusions

In this paper, a process monitoring framework, termed EAdspB-TLM, is proposed for monitoring nonlinear large-scale processes. When training data are insufficient to train a reliable model, traditional process monitoring methods cannot work well. As a result, faults of wastewater treatment and chemical processes cannot be identified and pre-alarmed in time, thus increasing the cost of system maintenance. Therefore, the proposed EAdspB-TLM was equipped with the ability of transfer learning, which allows useful information of unused data to be transferred to assist in training the model. EAdspB-TLM effectively alleviates the problem of insufficient label data in factories. Furthermore, the corresponding results also further verify the feasibility of the proposed EAdspB-TLM. According to the experimental results, with the increase of labeled target domain data, the diagnostic accuracy of EAdspB-TLM is improved. In addition, the pre-alarm rate (PAR) of EAdspB-TLM is also reduced. Overall, EAdspB-TLM achieved the best performance in monitoring the wastewater treatment and TE chemical processes. Using the WWTP as an example, when PR = 8%, the accuracy of the five methods can be ranked as follows: EAdspB-TLM (96.77%) > RVMt (92.47%) > SVM (90.32%) > PCA-T^2^ (84.95%) > RVM (68.82%).

The batch dataset needed for wastewater treatment process monitoring is drawn mostly from a collection of sensors. However, the data collected by some sensors has little value in training the monitoring model. Therefore, future research work will aim to optimize the number of selected sensors for monitoring and improve the monitoring efficiency of EAdspB-TLM.

## Figures and Tables

**Table 1:** Faults description of TECP.

No.	Description	Type
Fault 1	D feed temperature (stream 2)	Step
Fault 2	C header pressure loss-reduced availability (stream4)	Step
Fault 3	Condenser cooling water inlet temperature	Random
Fault 4	Reaction kinetics	Slow drift
Fault 5	Reactor cooling water valve	Sticking

**Table 2:** Monitoring results for Fault 1.

KPI		PCA-T^2^	SVM	RVM	RVMt	EAdspB-TLM
	3%	0.5179	0.0655	0.0179	0.1071	0.0565
FAR	5%	0.3894	0.0249	0.0561	0.1090	0.0654
	8%	0.3771	0.0337	0.0370	0.0606	0.0438
	3%	0.2842	0.7442	0.9044	0.2713	0.2222
MAR	5%	0.3272	0.8311	0.7361	0.2375	0.2190
	8%	0.3624	0.7847	0.7929	0.2452	0.1962
	3%	0.3777	0.4727	0.5498	0.2056	0.1560
PAR	5%	0.3521	0.5086	0.4641	0.1861	0.1576
	8%	0.3683	0.4843	0.4906	0.1714	0.1352
	3%	0.6072	0.5712	0.5076	0.8050	0.8548
Acc	5%	0.6443	0.5386	0.5757	0.8214	0.8514
	8%	0.6310	0.5512	0.5452	0.8373	0.8720

**Table 3:** Monitoring results for Fault 5.

KPI		PCA-T^2^	SVM	RVM	RVMt	EAdspB-TLM
	3%	0.5268	0.0952	0.1280	0.0149	0.0060
FAR	5%	0.2710	0.1246	0.2336	0.0187	0.0093
	8%	0.1380	0.0741	0.0606	0.0034	0.0067
	3%	0.3798	0.3514	0.5065	0.1680	0.1214
MAR	5%	0.5251	0.4274	0.3773	0.1451	0.1135
	8%	0.5395	0.4196	0.4033	0.0790	0.0654
	3%	0.4386	0.2489	0.3551	0.1067	0.0752
PAR	5%	0.4235	0.3063	0.3198	0.0945	0.0718
	8%	0.3789	0.2814	0.2662	0.0488	0.0419
	3%	0.5519	0.7676	0.6694	0.9032	0.9322
Acc	5%	0.5914	0.7114	0.6886	0.9129	0.9343
	8%	0.6401	0.7349	0.7500	0.9548	0.9608

**Table 4:** Average of false alarm rate (FAR) and missed alarm rate (MAR).

KPI (Average)		PCA-T^2^	SVM	RVM	RVMt	EAdspB-TLM
	3%	0.3929	0.3202	0.2161	0.0381	0.0220
FAR	5%	0.3327	0.1489	0.2617	0.0349	0.0218
	8%	0.4397	0.1906	0.0512	0.0189	0.0162
	3%	0.2739	0.3292	0.5447	0.1535	0.1323
MAR	5%	0.3203	0.5736	0.4121	0.1530	0.1277
	8%	0.3074	0.3591	0.4262	0.1341	0.0845

**Table 5:** Monitoring results for filamentous sludge bulking.

KPI		PCA-T^2^	SVM	RVM	RVMt	EAdspB-TLM
	3%	0.0877	0.0702	0.0000	0.1579	0.0175
FAR	5%	0.0357	0.0357	0.0000	0.1607	0.0179
	8%	0.1111	0.0185	0.0185	0.1296	0.0000
	3%	0.5366	0.4390	1.0000	0.0000	0.0732
MAR	5%	0.5610	0.4390	0.9512	0.0000	0.0732
	8%	0.2051	0.2051	0.7179	0.0000	0.0769
	3%	0.3570	0.2915	0.6000	0.0632	0.0509
PAR	5%	0.3509	0.2777	0.5707	0.0643	0.0510
	8%	0.1675	0.1305	0.4382	0.0519	0.0462
	3%	0.7245	0.7755	0.5816	0.9082	0.9592
Acc	5%	0.7423	0.7938	0.5979	0.9072	0.9588
	8%	0.8495	0.9032	0.6882	0.9247	0.9677
